# Choosing event‐driven and daily HIV pre‐exposure prophylaxis – data from two European PrEP demonstration projects among men who have sex with men

**DOI:** 10.1002/jia2.25768

**Published:** 2021-08-12

**Authors:** Vita W Jongen, Thijs Reyniers, Zorah MH Ypma, Maarten F Schim van der Loeff, Udi Davidovich, Hanne ML Zimmermann, Liza Coyer, Mark AM van den Elshout, Henry JC de Vries, Kristien Wouters, Tom Smekens, Bea Vuylsteke, Maria Prins, Marie Laga, Elske Hoornenborg

**Affiliations:** ^1^ Department of Infectious Diseases Public Health Service Amsterdam Amsterdam The Netherlands; ^2^ Department of Public Health Institute of Tropical Medicine Antwerp Belgium; ^3^ Internal Medicine Amsterdam Infection and Immunity (AII) Amsterdam UMC University of Amsterdam Amsterdam The Netherlands; ^4^ Department of Social Psychology University of Amsterdam Amsterdam The Netherlands; ^5^ Department of Dermatology Amsterdam Infection and Immunity (AII), location Academic Medical Centre Amsterdam UMC University of Amsterdam Amsterdam The Netherlands

**Keywords:** pre‐exposure prophylaxis, sexually transmitted infections, event‐driven PrEP, syphilis, men who have sex with men, HIV prevention & control

## Abstract

**Introduction:**

Daily and event‐driven PrEP are both efficacious in reducing the risk for HIV infection. However, the practice of event‐driven PrEP (edPrEP) is less well studied, in particular when provided as an alternative to daily PrEP. We studied regimen preferences and switches, and sexually transmitted infection (STI) incidence.

**Methods:**

We analysed pooled data from two prospective cohort studies among MSM: Be‐PrEP‐ared, Belgium and AMPrEP, the Netherlands. In both projects, participants could choose between daily and edPrEP at three‐monthly study visits, when they were also screened for sexually transmitted infections including hepatitis C (HCV). We assessed the proportion choosing each regimen, and the determinants of choosing edPrEP at baseline. Additionally, we compared the incidence rates (IRs) of HCV, syphilis and chlamydia or gonorrhoea between regimens using Poisson regression. The study period was from 3 August 2015 until 24 September 2018.

**Results and discussion:**

We included 571 MSM, of whom 148 (25.9%) chose edPrEP at baseline. 31.7% of participants switched regimen at least once. After 28 months, 23.5% used edPrEP. Older participants (adjusted odds ratio (aOR) = 1.38 per 10 years, 95% confidence interval (CI) = 1.15 to 1.64) and those unemployed (aOR = 1.68, 95% CI = 1.03 to 1.75) were more likely to initially choose edPrEP. IR of HCV and syphilis did not differ between regimens, but the IR of chlamydia/gonorrhoea was higher among daily users (adjusted incidence rate ratio = 1.61, 95% CI = 1.35 to 1.94).

**Conclusions:**

A quarter of participants chose edPrEP at baseline and at 28 months this proportion was similar. Although the IR of HCV and syphilis were similar in the two regimens, the lower incidence of chlamydia and gonorrhoea among edPrEP users may suggest that less frequent STI testing of this group could be considered.

## 1 Introduction

Oral pre‐exposure prophylaxis (PrEP) with tenofovir disoproxil fumarate and emtricitabine, is efficacious for human immunodeficiency virus (HIV) prevention, provided that it is taken correctly [1, 2, 3]. Among men who have sex with men (MSM), PrEP can be taken on a daily or event‐driven basis. The latter involves taking two pills two to twenty‐four hours before a sex act, followed by a daily tablet until 48 hours after the last sex act [2]. Offering a choice between these regimens may increase PrEP uptake, especially among individuals reluctant to take daily medication [4, 5, 6]. Additionally, as event‐driven PrEP (edPrEP) users take fewer tablets than daily users [7, 8], this regimen could reduce costs at individual and population levels and improve cost‐effectiveness [9, 10, 11]. The ANRS IPERGAY trial demonstrated the efficacy of edPrEP [2, 12] and the World Health Organization (WHO) recommended to include an option for edPrEP in PrEP programmes for MSM in 2019 [13]. However, edPrEP is currently not offered in most PrEP programmes worldwide. Only a limited number of studies have examined edPrEP [8, 14, 15, 16, 17, 18, 19]. To enable PrEP programme tailoring to the needs of all MSM, greater understanding of the characteristics of those choosing edPrEP is needed. The objective of this study was to assess regimen choice, switches between regimens and the incidence of HIV and sexually transmitted infections (STIs) using pooled data from MSM participating in two PrEP demonstration projects: Be‐PrEP‐ared (Antwerp, Belgium) [18, 20] and AMPrEP (Amsterdam, the Netherlands) [19, 21, 22].

## 2 Methods

### 2.1 Study design and participants

We used pseudonymized pooled data from the Be‐PrEP‐ared and AMPrEP studies. Pooling data enabled us to study a relatively large number of edPrEP users and thus increase statistical power. Full procedures of both studies have been published previously [18, 19, 20, 22]. In brief, AMPrEP enrolled participants between August 2015 and June 2016; Be‐PrEP‐ared between October 2015 and December 2016. In both studies HIV‐negative MSM and transgender persons were eligible for inclusion if they were ≥18 years old and if they reported any of the following in the previous six months: condomless anal sex with casual partners, at least one diagnosed bacterial STI, post‐exposure prophylaxis (PEP) use, or sex with a partner living with HIV with an unknown or detectable viral load. As the WHO advises to offer edPrEP to cis‐men only [13], we excluded the transgender persons (n = 5) from this analysis. At baseline, participants were offered a choice between daily and edPrEP and they could switch between regimens at every three‐monthly study visit. Study staff explained both regimens to the participants, after which they could ask questions and choose their preferred regimen. Daily users were provided with three boxes containing 30 PrEP tablets. The number of boxes provided to edPrEP users was based on their expected needs and the number of pills they had left. Generally, this meant edPrEP users were provided with one or two boxes containing 30 tablets. The Be‐PrEP‐ared and AMPrEP studies ended in May 2018 and December 2020 respectively. For the current analysis, the data were censored for all participants at 28 months after the date of inclusion (i.e. the longest possible follow‐up time in the Be‐PrEP‐ared). Thus, the study period for this study was from 3 August 2015 to 24 September 2018.

### 2.2 Procedures

At baseline, participants completed self‐administered computer‐assisted questionnaires with questions on socio‐demographic characteristics (e.g. education) and sexual behaviour.

Details of STI screening and laboratory procedures have been described previously [22, 23, 24]. In short, participants were screened for HIV, syphilis and urogenital, anal and pharyngeal chlamydia and gonorrhoea at three‐monthly study visits [20, 22]. Additional STI testing between three‐monthly study visits was possible and those results were included in the analysis. Free‐of‐charge treatment was provided to participants diagnosed with an STI.

In the Be‐PrEP‐ared study, hepatitis C (HCV) antibodies (anti‐HCV) were initially tested at the screening visit and after 18 months of follow‐up. HCV testing frequency was increased to six‐monthly starting May 2017 [20]. HCV testing in AMPrEP (anti‐HCV and HCV RNA if antibodies were present) was performed every 12 months until December 2016, after which testing was done bi‐annually [24, 25]. Additional HCV testing in AMPrEP was performed in case of partner notification or clinical indication.

### 2.3 Statistical analysis

Baseline demographics and PrEP choice were compared between Be‐PrEP‐ared and AMPrEP using rank sum tests for continuous variables and Pearson’s *χ* ^2^ or Fisher’s exact tests for categorical variables. Determinants for choosing edPrEP at baseline were assessed using logistic regression with a random‐intercept to account for between‐city variability. In multivariable analysis we included determinants associated in univariable analysis at *p* < 0.20 (Wald test). Backwards selection was performed to obtain a parsimonious multivariable model.

Incidence rates (IRs) of HIV, HCV, syphilis, chlamydia and gonorrhoea per 100 person‐years were estimated by dividing the number of incident infections by person‐years of observation. We defined an incident HCV infection as the first newly diagnosed HCV infection (anti‐HCV positive and/or HCV RNA positive) among participants who were HCV‐negative at baseline (anti‐HCV negative and/or HCV RNA negative). Repeated HCV infections were not included in the analyses because Be‐PrEP‐ared only determined anti‐HCV and hence reinfections could not be identified. For infectious syphilis (stage 1 or 2), chlamydia and gonorrhoea repeated infections over time were included in the analysis. In case of an incident HIV or HCV infection, we assumed the infection occurred at midpoint between the last negative and the first positive test, after which time at‐risk stopped. All follow‐up time was considered time at risk for syphilis, chlamydia and gonorrhoea. IRs were estimated for the overall study population and stratified by PrEP regimen used at the moment of STI diagnosis. Incidence rate ratios (IRRs), adjusted for age and testing frequency per total months in follow‐up (as participants who test more frequently have a higher chance of getting a positive STI diagnosis), were estimated using Poisson regression with a random‐intercept to account for between‐city variability.

All analyses were performed using Stata (version 15.1, StataCorp, College Station, TX, USA).

## 3 Results and discussion

We included 571 MSM in the analysis, 197 (34.5%) from Be‐PrEP‐ared and 374 from AMPrEP (65.5%). Median age was 39 years in both studies (Table 1). At baseline, AMPrEP participants were less likely to be exclusively homosexual (91.2% vs. 97.5%, *p* = 0.004), less often had a STI in the six months prior (36.1% vs. 58.9%, *p* < 0.001), and less often had used PEP in the six months prior (7.2% vs. 14.7%, *p* = 0.004).

**Table 1:** Baseline characteristics of 571 men who have sex with men participating in AMPrEP (Amsterdam, The Netherlands) and Be‐PrEP‐ared (Antwerp, Belgium) cohort studies, August 2015 to December 2016

	Total (n = 571)		AMPrEP (n = 374)		Be‐PrEP‐ared (n = 197)		*p*‐value^b^
	n^a^	%^a^	n^a^	%^a^	n^a^	%^a^	
PrEP regimen							
Daily	423	74.1	272	72.7	151	76.7	0.31
Event‐driven	148	25.9	102	27.3	46	23.4	
Age (in years)							
Median [IQR]	39	[32 to 47]	39	[32 to 48]	39	[32 to 44]	0.23
<35	189	33.1	126	33.7	63	32.0	0.001
35‐44	199	34.9	112	30.0	87	44.2	
≥45	183	32.1	136	36.4	47	23.9	
Self‐declared racial‐ethnic background							
White	494	86.5	319	85.3	175	88.8	0.24
Other	77	13.5	55	14.7	22	11.2	
Highest education level^c^							
No college/university	129	22.7	87	23.5	42	21.3	0.56
College/university	439	77.3	284	76.6	155	78.7	
Employment^d^							
Employed^e^	477	84.1	311	84.1	166	84.3	0.95
Unemployed/other^f^	90	15.9	59	16.0	31	15.7	
Living situation							
Alone	295	51.7	198	52.9	97	49.2	0.82
With steady partner	187	32.8	121	32.4	66	33.5	
With parents	18	3.2	11	2.9	7	3.6	
With roommates	71	12.4	44	11.8	27	13.7	
Sexual preference^g^							
Not exclusively homosexual	38	6.7	33	8.9	5	2.5	0.004
Exclusively homosexual	532	93.3	340	91.2	192	97.5	
In a steady relationship^d^							
No	313	55.2	206	55.7	107	54.3	0.76
Yes	254	44.8	164	44.3	90	45.7	
Sexually transmitted infection (6 months)^h^, ^i^							
No	320	56.0	239	63.9	81	41.1	<0.001
Yes	251	44.0	135	36.1	116	58.9	
CAS with casual partner(s) (6 months)^h^							
No	24	4.2	17	4.6	7	3.6	0.57
Yes	547	95.8	357	95.5	190	96.5	
PEP used (6 months)^h^							
No	515	90.2	347	92.8	168	85.3	0.004

CAS, condomless anal sex; IQR, interquartile range; PEP, post‐exposure prophylaxis; PrEP, pre‐exposure prophylaxis

^a^ Unless specified otherwise

^b^ continuous variables were compared using a Wilcoxon rank‐sum test. Categorical variables were compared using a Pearson’s chi‐squared test or Fisher’s Exact test

^c^ 3 missing (AMPrEP)

^d^ 4 missing (AMPrEP)

^e^ both full‐time and part‐time employed

^f^ other includes volunteer work, being retired and being a student, being unable to work due to disability

^g^ 1 missing (AMPrEP)

^h^ in the six months before baseline

^i^ at least one bacterial sexually transmitted infection (i.e. syphilis, or urethral or rectal chlamydia or gonorrhoea).

### 3.1 Preferences of PrEP regimen

At baseline, 148 (25.9%) participants chose edPrEP (Table 1). In multivariable analysis, older age (adjusted OR (aOR) 1.36 per 10‐year increase in age, 95% confidence interval (CI) 1.14 to 1.63) and being unemployed (aOR 1.72, 95% CI 1.05 to 2.82) increased the odds of choosing edPrEP at baseline (Table 2).

**Table 2:** Univariable and multivariable analysis of determinants for choosing the event‐driven PrEP regimen in the AMPrEP (Amsterdam, The Netherlands) and Be‐PrEP‐ared (Antwerp, Belgium) cohort studies (N = 571), August 2015 to December 2016

	Total	Daily PrEP	Event‐driven PrEP	Univariable logistic regression			Multivariable logistic regression		
	n (%)^a^	n (%)^a^	n (%)^a^	OR	(95% CI)	*p*‐value	aOR^b^	(95% CI)	*p*‐value
Age per 10 year increase, median [IQR]	3.9 [3.2 to 4.7]	3.8 [3.1 to 4.6]	4.3 [3.5 to 5.0]	1.43	(1.20 to 1.70)	<0.001	1.36	(1.14 to 1.63)	0.001
Age (categorical)						0.007			
<35 years	189 (33.1%)	152 (35.9%)	37 (25.0%)	REF					
35‐44 years	199 (34.8%)	150 (35.5%)	49 (33.1%)	1.34	(0.83 to 2.17)				
≥45 years	183 (32.1%)	121 (28.6%)	62 (41.9%)	2.10	(1.31 to 3.37)				
Self‐declared racial‐ethnic background						0.17			
White	494 (86.5%)	361 (85.3%)	133 (89.9%)	REF					
Other	77 (13.5%)	62 (14.7%)	15 (10.1%)	0.66	(0.36 to 1.19)				
Highest education level^c^						0.29			
No college/university	129 (22.7%)	100 (23.8%)	29 (19.6%)	REF					
College/university	439 (77.3%)	320 (76.2%)	119 (80.4%)	1.28	(0.81 to 2.04)				
Employment^d^						0.003			
Employed^e^	477 (84.1%)	364 (86.9%)	113 (76.4%)	REF			REF		
Unemployed and other^f^	90 (15.9%)	55 (13.1%)	35 (23.7%)	2.05	(1.28 to 3.29)		1.72	(1.05 to 2.82)	0.032
Living situation						0.50			
Alone	295 (51.7%)	212 (50.1%)	83 (56.1%)	REF					
With steady partner	187 (32.8%)	140 (33.1%)	47 (31.8%)	0.86	(0.57 to 1.30)				
With parents	18 (3.2%)	14 (3.3%)	4 (2.7%)	0.73	(0.23 to 2.28)				
With roommates	71 (12.4%)	57 (13.5%)	14 (9.5%)	0.63	(0.33 to 1.19)				
Sexual preference^g^						0.29			
Not exclusively homosexual	38 (6.7%)	31 (7.3%)	7 (4.8%)	REF					
Exclusively homosexual	532 (93.3%)	392 (92.7%)	140 (95.2%)	1.58	(0.68 to 3.67)				
In a steady relationship^d^						0.42			
No	313 (55.2%)	236 (56.2%)	77 (52.4%)	REF					
Yes	254 (44.8%)	184 (43.8%)	70 (47.6%)	1.17	(0.80 to 1.70)				
Sexually transmitted infection (6 months)^h^, ^i^						0.05			
No	320 (56.0%)	227 (53.7%)	93 (62.8%)	REF					
Yes	251 (44.0%)	196 (46.3%)	55 (37.2%)	0.68	(0.47 to 1.01)				
CAS with casual partner(s) (6 motnhs)^h^						0.40			
No	24 (4.2%)	16 (3.8%)	8 (5.4%)	REF					
Yes	547 (95.8%)	407 (96.2%)	140 (94.6%)	0.69	(0.29 to 1.64)				
PEP use (6 months)^h^						0.42			
No	515 (90.2%)	379 (89.6%)	136 (91.9%)	REF					
Yes	56 (9.8%)	44 (10.4%)	12 (8.1%)	0.76	(0.39 to 1.48)				

aOR, adjusted Odds Ratio; CAS, condomless anal sex; CI, confidence interval; IQR, interquartile range; OR, odds ratio; PEP, post‐exposure prophylaxis; PrEP, pre‐exposure prophylaxis

^a^ Unless otherwise indicated

^b^ multivariable logistic regression model based on 567 participants

^c^ 3 missing (AMPrEP)

^d^ 4 missing (AMPrEP)

^e^ both full‐time and part‐time employed

^f^ other includes volunteer work, being retired, being a student and being unable to work due to disability

^g^ 1 missing (AMPrEP)

^h^ in the six months before baseline

^i^ at least one bacterial sexually transmitted infection (i.e. syphilis, or urethral or rectal chlamydia or gonorrhoea).

Of the 571 enrolled participants, 13 had no follow‐up data. Median follow‐up time was 26 months [IQR 21 to 27]. Three hundred eighty‐one participants (68.3%) never switched between PrEP regimens, 96 (17.2%) switched once and 81 (14.5%) more than once. In total, 228 switches were reported, of which 153 (53.1%) were from the daily to the edPrEP regimen. After 28 months, 23.5% of participants used edPrEP.

### 3.2 STI prevalence and incidence

Two participants acquired HIV during follow‐up; both were using the daily PrEP regimen. One stopped taking PrEP [22], whereas the other participant consistently took PrEP (as indicated by tenofovir diphosphate concentrations from dried blood spots [26]). Overall HIV IR was 0.2 per 100 person‐years (95% CI 0.1 to 0.8).

At baseline, 21 participants had evidence of current or past HCV infection (prevalence 3.7%, 95% CI 2.3 to 5.6%). Seventeen new HCV infections were diagnosed during 993.9 person‐years of follow‐up (IR 1.7 per 100 person‐years, 95% CI 1.1 to 2.8). HCV IR did not differ between periods of daily and event‐driven use (aIRR 1.03, 95% CI 0.33 to 3.18) (Table 3).

**Table 3:** Incidence rate of hepatitis C, syphilis, chlamydia and gonorrhoea by PrEP regimen, and the incidence rate ratio comparing PrEP regimens, in the AMPrEP (Amsterdam, The Netherlands) and Be‐PrEP‐ared (Antwerp, Belgium) cohort studies (N = 558); August 2015 to September 2018

	Daily regimen					Event‐driven regimen					Daily versus event‐driven		
	n^a^	Incident infections	Person‐years	IR^b^	(95% CI)	n^a^	Incident infections	Person‐years	IR^b^	(95% CI)	aIRR^c^	(95% CI)	*p*‐value
Hepatitis C^d^	467	13	747.7	1.7	(1.0 to 3.0)	235	4	246.3	1.6	(0.6 to 4.3)	1.03	(0.33 to 3.18)	0.96
Syphilis	484	86	759.4	11.3	(9.2 to 14.0)	247	30	259.5	11.6	(8.1 to 16.5)	0.90	(0.59 to 1.37)	0.63
Any chlamydia or gonorrhoea^e^	484	709	788.2	90.0	(83.6 to 96.8)	247	140	269.9	51.9	(44.0 to 61.2)	1.61	(1.35 to 1.94)	<0.001
Any anal chlamydia or gonorrhoea^e^	484	513	788.2	65.1	(59.7 to 71.0)	247	94	269.9	34.8	(28.5 to 42.6)	1.74	(1.39 to 2.16)	<0.001
Any chlamydia	484	370	788.2	46.9	(42.4 to 52.0)	247	77	269.9	28.5	(22.8 to 35.7)	1.54	(1.20 to 1.96)	0.001
Anal chlamydia	484	277	788.2	35.1	(31.2 to 39.5)	247	54	269.9	20.0	(15.3 to 26.1)	1.65	(1.23 to 2.21)	0.001
Urogenital chlamydia	484	100	788.2	12.7	(10.4 to 15.4)	247	26	269.9	9.6	(6.6 to 14.2)	1.25	(0.81 to 1.93)	0.31
Pharyngeal chlamydia	484	37	788.2	4.7	(3.4 to 6.5)	247	8	269.9	3.0	(1.5 to 5.9)	1.44	(0.67 to 3.11)	0.35
Anal LGV	484	43	788.2	5.5	(4.0 to 7.4)	247	5	269.9	1.9	(0.8 to 4.5)	2.82	(1.11 to 7.13)	0.029
Any gonorrhoea	484	436	788.2	55.3	(50.4 to 60.8)	247	83	269.9	30.8	(24.8 to 38.1)	1.68	(1.32 to 2.11)	<0.001
Anal gonorrhoea	484	299	788.2	37.9	(33.9 to 42.5)	247	52	269.9	19.3	(14.7 to 25.3)	1.84	(1.37 to 2.48)	<0.001
Urogenital gonorrhoea	484	80	788.2	10.1	(8.2 to 12.6)	247	12	269.9	4.4	(2.5 to 7.8)	2.19	(1.19 to 4.04)	0.012
Pharyngeal gonorrhoea	484	196	788.2	24.9	(21.6 to 28.6)	247	43	269.9	15.9	(11.8 to 21.5)	1.42	(1.02 to 1.98)	0.036

aIRR, adjusted incidence rate ratio; CI, confidence interval; IR, incidence rate; LGV, lymphogranuloma venereum

^a^ As participants could switch at every three‐monthly study visit, participants could add person‐time to both regimens

^b^ per 100 person‐years

^c^ adjusted for age and testing frequency per total months in follow‐up

^d^ participant who had evidence of a current or past HCV infection at baseline (n = 21) were excluded from this analysis

^e^ the diagnosis of both chlamydia and gonorrhoea at a study visit was counted as one incident event.

Twenty syphilis infections were found at baseline (prevalence 3.6%, 95% CI 2.2 to 5.5%). During 1018.9 person‐years, 116 new syphilis infections were diagnosed (IR 11.4 per 100 person‐years, 95% CI 9.5 to 13.7). Syphilis IR did not differ between periods of daily and event‐driven use (aIRR 0.90, 95% CI 0.59 to 1.37).

During follow‐up, 368 participants (65.9%) were diagnosed with one or more incident chlamydia or gonorrhoea infections. Overall IR of any chlamydia or gonorrhoea was 80.2 per 100 person‐years (95% CI 75.0 to 85.8). Incidence of any chlamydia or gonorrhoea was 61% higher during daily compared to event‐driven use (aIRR 1.61, 95% CI 1.35 to 1.94) (Table 3).

### 3.3 Discussion

We showed that some MSM prefer edPrEP over daily PrEP, indicating there is a need for a choice in PrEP programmes, although the proportion MSM who chose edPrEP varies between studies. EdPrEP uptake in our study was 26%, compared to 19% in the Canadian l’Actual PrEP cohort study (19%) [16] and 16% among individuals buying PrEP online in the UK [17]. In contrast, edPrEP uptake was higher among patients of a clinic in Paris, France (76%) [14]. As this clinic was involved in the ANRS IPERGAY trial [2], clinicians and patients were probably more familiar with edPrEP, leading to higher uptake. EdPrEP uptake was also high in national PrEP programmes in 2019: 56% of 1654 new Belgian PrEP users chose the edPrEP regimen [27], while in the Netherlands edPrEP use was reported in 39% of 1837 consultations [28]. In our study older participants were more likely to choose edPrEP, similar to observations from other studies [14, 16]. Older MSM may be more likely to have less sexual encounters and therefore are more motivated to choose edPrEP [6]. Additionally, we found that being unemployed increased the odds of choosing edPrEP, contrary to what was previously observed [14]. The reason for this should be further explored.

HIV incidence was low, consistent with previous PrEP studies among MSM [1]. No seroconversions were diagnosed during edPrEP use, building on the evidence that edPrEP is highly effective against HIV [2, 8, 12]. We found that the incidence of chlamydia and gonorrhoea was over 60% higher during daily use. This is potentially due to fewer condomless sex acts among edPrEP users [20, 21, 22]. While the current PrEP guidelines of the Netherlands [29] and Belgium [30] advise to test for STIs four times a year, our results suggest that testing less frequently for chlamydia and gonorrhoea among edPrEP users may be possible, albeit highly dependent on sexual behaviour. As the incidence of HCV and syphilis did not differ between daily and edPrEP users, three‐monthly screening for these STIs remains advisable.

The following limitations should be taken into account. First, we only determined HCV‐antibodies in Be‐PrEP‐ared and hence could not include HCV reinfections in our analysis. In addition, although we included extra STI screening visits at the STI clinics between scheduled study visits, participants might have had additional STI visits outside of the study centres, so reported STI IRs may be underestimates. Second, the majority of participants were white and highly educated, thus results are not generalizable to the entire MSM population. Third, when both studies started, edPrEP was less well known, and not yet included in the WHO guideline [13]. This may have affected the choice for edPrEP. Last, reasons for switching between PrEP regimens were assessed separately for AMPrEP [6, 31] and Be‐PrEP‐ared [20], but these data were not included and further analysed in this pooled analysis.

## 4 Conclusions

A quarter of participants chose edPrEP at baseline. Although switching between regimens was common, the proportion of participants using edPrEP was similar at 28 months. PrEP programmes should enable individuals to adapt their PrEP use to their needs (e.g. by providing more options than daily PrEP and giving information on how to safely switch regimens). The lower incidence of chlamydia and gonorrhoea among edPrEP users may suggest that less frequent STI testing of this group could be considered, even though the IRs of HCV and syphilis were similar. Further research on proportions and characteristics of edPrEP users and optimal STI testing frequency is warranted.

## Competing interest

The study medication for the Be‐PrEP‐ared study of Antwerp and the Amsterdam PrEP study was provided by Gilead Sciences based on unconditional grants. EH received advisory board fees from Gilead Sciences and speaker fees from Janssen‐Cilag, both paid to her institute. UD received unrestricted research grants and speaker’s fees from Gilead Sciences, paid to his institute. HJCdV received grants from Medigene, and advisory board and speaker fees from Gilead Sciences, Medigene, Abbvie, Janssen‐Cilag and Willpharma, paid to his institute. MP received unrestricted research grants and speaker’s fees from Gilead Sciences, Roche, Abbvie and MSD, paid to her institute. MSvdL served on an Advisory Board of MSD, paid to his institute. All other authors declare no competing interest.

## Authors’ contributions

BV, TR, EH, MP, ML, MSvdL, HdV and UD conceptualized and designed the Tale of Two Cities project and obtained funding. ZY, VJ and MSvdL were involved in the data analysis. VJ, TR, ZY, MSvdL, UD, HZ, LC, MvdE, HdV, KW, TS, BV, MP, ML and EH were involved with interpretation of the data. VJ drafted the manuscript. All authors read and approved the final manuscript.

## Ethical considerations

The Be‐PrEP‐ared study (EudraCT 2015‐000054‐37) was approved by the institutional review board of the Institute of Tropical Medicine, Antwerp (988/15), and the ethics committee of the Antwerp University Hospital (15/25/255). The AMPrEP study obtained ethical approval from the ethics board of the Academic Medical Center, Amsterdam, the Netherlands (NL49504.018.14). AMPrEP was registered at the online Dutch trial registry (NTR5411). All participants provided written informed consent.

## References

[1] Fonner VA, Dalglish SL, Kennedy CE, et al. Effectiveness and safety of oral HIV preexposure prophylaxis for all populations. AIDS. 2016;30:1973–83. 27149090 10.1097/QAD.0000000000001145 PMC4949005

[2] Molina JM, Capitant C, Spire B, et al. On‐demand preexposure prophylaxis in men at high risk for HIV‐1 infection. N Engl J Med. 2015;373:2237–46. 26624850 10.1056/NEJMoa1506273

[3] McCormack S, Dunn DT, Desai M, et al. Pre‐exposure prophylaxis to prevent the acquisition of HIV‐1 infection (PROUD): effectiveness results from the pilot phase of a pragmatic open‐label randomised trial. Lancet. 2016;387:53–60. 26364263 10.1016/S0140-6736(15)00056-2 PMC4700047

[4] Hojilla JC, Marcus JL, Silverberg MJ, et al. Early adopters of event‐driven human immunodeficiency virus pre‐exposure prophylaxis in a large healthcare system in San Francisco. Clin Infect Dis. 2020;71(10):2710–12. 10.1093/cid/ciaa474 32494806 PMC7744988

[5] Cornelisse VJ, Lal L, Price B, et al. Interest in switching to on‐demand hiv pre‐exposure prophylaxis (PrEP) among Australian users of daily PrEP: an online survey. open forum. Infect Dis. 2019;6:ofz287. 10.1093/ofid/ofz287 PMC6612821 31304192

[6] Zimmermann HM, Eekman SW, Achterbergh RC, et al. Motives for choosing, switching and stopping daily or event‐driven pre‐exposure prophylaxis – a qualitative analysis. J Int AIDS Soc. 2019;22:e25389. 31612621 10.1002/jia2.25389 PMC6791997

[7] Finkenflugel RNN, Hoornenborg E, Achterbergh RCA, et al. A mobile application to collect daily data on pre‐exposure prophylaxis adherence and sexual behaviour among men who have sex with men: use over time and comparability with conventional data collection. Sex Transm Dis. 2019;46:400–6. 30882717 10.1097/OLQ.0000000000000999 PMC6553988

[8] Molina J‐M, Charreau I, Spire B, et al. Efficacy, safety, and effect on sexual behaviour of on‐demand pre‐exposure prophylaxis for HIV in men who have sex with men: an observational cohort study. Lancet HIV. 2017;4:e402–10. 28747274 10.1016/S2352-3018(17)30089-9

[9] Ouellet E, Durand M, Guertin JR, LeLorier J, Tremblay CL. Cost effectiveness of 'on demand' HIV pre‐exposure prophylaxis for non‐injection drug‐using men who have sex with men in Canada. Can J Infect Dis Med Microbiol. 2015;26:23–9. 25798150 10.1155/2015/964512 PMC4353265

[10] Nichols BE, Boucher CAB, van der Valk M, Rijnders BJA, van de Vijver DAMC. Cost‐effectiveness analysis of pre‐exposure prophylaxis for HIV‐1 prevention in the Netherlands: a mathematical modelling study. Lancet Infect Dis. 2016;16:1423–9. 27665989 10.1016/S1473-3099(16)30311-5

[11] Durand‐Zaleski I, Mutuon P, Charreau I, et al. Costs and benefits of on‐demand HIV preexposure prophylaxis in MSM. AIDS. 2018;32:95–102. 29210777 10.1097/QAD.0000000000001658

[12] Antoni G, Tremblay C, Delaugerre C, et al. On‐demand pre‐exposure prophylaxis with tenofovir disoproxil fumarate plus emtricitabine among men who have sex with men with less frequent sexual intercourse: a post‐hoc analysis of the ANRS IPERGAY trial. Lancet HIV. 2020;7:e113–20. 31784343 10.1016/S2352-3018(19)30341-8

[13] WHO . What’s the 2+1+1? Event‐driven oral pre‐exposure prophylaxis to prevent HIV for men who have sex with men: update to WHO’s recommendation on oral PrEP. Geneva: World Health Organization; 2019.

[14] Noret M, Balavoine S, Pintado C, et al. Daily or on‐demand oral tenofovir disoproxil fumarate/emtricitabine for HIV pre‐exposure prophylaxis: experience from a hospital‐based clinic in France. AIDS. 2018;32:2161–9. 30212403 10.1097/QAD.0000000000001939

[15] Grant RM, Mannheimer S, Hughes JP, et al. Daily and nondaily oral preexposure prophylaxis in men and transgender women who have sex with men: the human immunodeficiency virus prevention Trials network 067/ADAPT study. Clin Infect Dis. 2018;66:1712–21. 29420695 10.1093/cid/cix1086 PMC5961078

[16] Greenwald ZR, Maheu‐Giroux M, Szabo J, et al. Cohort profile: l'Actuel Pre‐Exposure Prophylaxis (PrEP) cohort study in Montreal, Canada. BMJ Open. 2019;9:e028768. 10.1136/bmjopen-2018-028768 PMC6597624 31248931

[17] Wang X, Nwokolo N, Korologou‐Linden R, et al. InterPrEP: internet‐based pre‐exposure prophylaxis with generic tenofovir disoproxil fumarate/emtrictabine in London – analysis of pharmacokinetics, safety and outcomes. HIV Med. 2018;19:1–6. 28657199 10.1111/hiv.12528

[18] Reyniers T, Nöstlinger C, Laga M, et al. Choosing between daily and event‐driven pre‐exposure prophylaxis: results of a belgian PrEP demonstration project. J Acquir Immune Defic Syndr. 2018;79:186–94. 29975211 10.1097/QAI.0000000000001791

[19] Hoornenborg E, Achterbergh RC, van der Loeff MFS, et al. Men who have sex with men more often chose daily than event‐driven use of pre‐exposure prophylaxis: baseline analysis of a demonstration study in Amsterdam. J Int AIDS Soc. 2018;21:e25105. 29603900 10.1002/jia2.25105 PMC5878413

[20] Vuylsteke B, Reyniers T, De Baetselier I, et al. Daily and event‐driven pre‐exposure prophylaxis for men who have sex with men in Belgium: results of a prospective cohort measuring adherence, sexual behaviour and STI incidence. J Int AIDS Soc. 2019;22:e25407. 31663257 10.1002/jia2.25407 PMC6819896

[21] Hoornenborg E, Coyer L, van Laarhoven A, et al. Change in sexual risk behaviour after 6 months of pre‐exposure prophylaxis use: results from the Amsterdam pre‐exposure prophylaxis demonstration project. AIDS. 2018;32:1527–32. 29762169 10.1097/QAD.0000000000001874

[22] Hoornenborg E, Coyer L, Achterbergh RCA, et al. Sexual behaviour and incidence of HIV and sexually transmitted infections among men who have sex with men using daily and event‐driven pre‐exposure prophylaxis in AMPrEP: 2 year results from a demonstration study. Lancet HIV. 2019;6:e447–55. 31178284 10.1016/S2352-3018(19)30136-5

[23] De Baetselier I, Reyniers T, Nöstlinger C, et al. Pre‐Exposure prophylaxis (PrEP) as an additional tool for HIV prevention among men who have sex with men in Belgium: the Be‐PrEP‐ared study protocol. JMIR Res Protoc. 2017;6:e11. 28135199 10.2196/resprot.6767 PMC5306610

[24] Hoornenborg E, Coyer L, Boyd A, et al. High incidence of HCV in HIV‐negative men who have sex with men using pre‐exposure prophylaxis. J Hepatol. 2019;72:855–64. 31862485 10.1016/j.jhep.2019.11.022

[25] Hoornenborg E, Achterbergh RCA, Schim van der Loeff MF, et al. MSM starting preexposure prophylaxis are at risk of hepatitis C virus infection. AIDS. 2017;31:1603–10. 28657964 10.1097/QAD.0000000000001522

[26] Hoornenborg E, Prins M, Achterbergh RCA, et al. Acquisition of wild‐type HIV‐1 infection in a patient on pre‐exposure prophylaxis with high intracellular concentrations of tenofovir diphosphate: a case report. Lancet HIV. 2017;4:e522–8. 28919303 10.1016/S2352-3018(17)30132-7

[27] Sasse A, Deblonde J, De Rouck M, Montourcy M, Van Beckhoven D. Epidemiologie van aids en hiv‐infectie in België. In: SCIENSANO, ed. 2020. https://www.sciensano.be/nl/biblio/epidemiologie‐van‐aids‐en‐hiv‐infectie‐belgie‐toestand‐op‐31‐december‐2019. Accessed 24 Dec 2020.

[28] Staritsky LE, Van Aar F, Visser M, et al. Sexually transmitted infections in the Netherlands in 2019. RIVM report 2020‐0052. 2020.

[29] Nederlandse Vereniging van HIV Behandelaren (NVHB) . HIV preexpositie profylaxe (PrEP) richtlijn Nederland, gereviseerde versie 2. Nederlandse vereniging van hiv behandelaren (NVHB). https://nvhb.nl/wp‐content/uploads/2019/04/PrEP‐richtlijn‐Nederland‐versie‐2‐dd‐15‐april‐2019.pdf. Accessed 15 Apr 2020.

[30] Rijksinstituut voor ziekte‐ en invaliditeitsverzekering . Geneesmiddel in PrEp om een HIV‐infectie te voorkomen: terugbetaling vanaf 1 juni 2017. https://www.inami.fgov.be/nl/themas/kost‐terugbetaling/door‐ziekenfonds/geneesmiddel‐gezondheidsproduct/terugbetalen/specialiteiten/wijzigingen/Paginas/geneesmiddelen‐PrEp‐HIV.aspx. Accessed 27 May 2021.

[31] Coyer L, van den Elshout MAM, Achterbergh RCA, et al. Understanding pre‐exposure prophylaxis (PrEP) regimen use: switching and discontinuing daily and event‐driven PrEP among men who have sex with men. EClinicalMedicine. 2020;29‐30:100650. 10.1016/j.eclinm.2020.100650 PMC7711206 33305198

